# DPP4 Deficiency Exerts Protective Effect against H_2_O_2_ Induced Oxidative Stress in Isolated Cardiomyocytes

**DOI:** 10.1371/journal.pone.0054518

**Published:** 2013-01-24

**Authors:** Hui-Chun Ku, Wen-Pin Chen, Ming-Jai Su

**Affiliations:** Institute of Pharmacology, College of Medicine, National Taiwan University, Taipei, Taiwan; Bristol Heart Institute, University of Bristol, United Kingdom

## Abstract

Apart from the antihyperglycemic effects, DPP4 inhibitors and GLP-1 molecules are involved in the preservation of cardiac functions. We have demonstrated that DPP4-deficient rats possess resistance to endotoxemia and ischemia/reperfusion stress. However, whether the decrease of DPP4 activity simply augmented the GLP-1 signaling or that such decrease resulted in a change of cellular function remain unclear. Accordingly, we investigated the responses of H_2_O_2_-induced oxidative stress in adult wild-type and DPP4-deficient rats isolated cardiomyocytes. The coadministration of GLP-1 or DPP4 inhibitor was also performed to define the mechanisms. Cell viability, ROS concentration, catalase activity, glucose uptake, prosurvival, proapoptotic signaling, and contractile function were examined after cells exposed to H_2_O_2_. DPP4-deficient cardiomyocytes were found to be resistant to H_2_O_2_-induced cell death via activating AKT signaling, enhancing glucose uptake, preserving catalase activity, diminishing ROS level and proapoptotic signaling. GLP-1 concentration-dependently improved cell viability in wild-type cardiomyocyte against ROS stress, and the ceiling response concentration (200 nM) was chosen for studies. GLP-1 was shown to decrease H_2_O_2_-induced cell death by its receptor-dependent AKT pathway in wild-type cardiomyocytes, but failed to cause further activation of AKT in DPP4-deficient cardiomyocytes. Acute treatment of DPP4 inhibitor only augmented the protective effect of low dose GLP-1, but failed to alter fuel utilization or ameliorate cell viability in wild-type cardiomyocytes after H_2_O_2_ exposure. The improvement of cell viability after H_2_O_2_ exposure was correlated with the alleviation of cellular contractile dysfunction in both DPP4-deficient and GLP-1 treated wild-type cardiomyocytes. These findings demonstrated that GLP-1 receptor-dependent pathway is important and exert protective effect in wild-type cardiomyocyte. Long term loss of DPP4 activity increased the capability against ROS stress, which was more than GLP-1 dependent pathway.

## Introduction

Dipeptidyl peptidase-4 (DPP4) cleaves multiple peptide substrates, including the incretin hormones glucagon-like peptide-1 (GLP-1) that stimulate insulin secretion from β-cells and inhibit hepatic glucose production [1]. A number of studies have demonstrated cytoprotective actions of GLP-1 in several kinds of cell type beyond its modulation of glucose metabolism [2]. GLP-1 inhibits cell apoptosis or necrosis in pancreatic βcells [3], neurons [4], endothelial cells [5], and cardiomyocytes [2]. Incubation with GLP-1 inhibits activation of apoptotic process and increases viability in neonatal cardiomyocytes undergoing hypoxia/reoxygenation injury [6]. GLP-1 also prevents activation of cell death signal in adult murine HL-1 cardiomyocytes incubated with staurosporine, a apoptotic stimuli [2]. In addition, the presence of GLP-1 signaling has been demonstrated in cardiac function preservation in various animal model experiments, such as dilated cardiomyopathy, heart failure, and myocardial infarction [1], [7], [8], [9], [10], [11], [12], [13]. All these experiments demonstrate that the cytoprotective effect of GLP-1 is mediated mainly by mechanisms dependent on the activation of the phosphatidylinositol 3-kinase (PI3K) and extracellular signal regulated kinase (ERK). Furthermore, it is worth noticing that several clinical studies showed the cardioprotective effects of GLP-1-based therapy against ischemic and failing hearts [8], [10], [14], [15].

In contrast to GLP-1, much less is known about the cardiovascular biology of DPP4. DPP4 known as CD26 is a homodimeric type II transmembrane glycoprotein, which is one of the accessory molecules of helper T cells, and has three major functions: adenosine deaminase binding, extracellular matrix binding, and peptidase activity [16], [17], [18]. A previous study showed that genetic deletion or pharmacological inhibition of DPP4 improved cardiovascular outcomes following myocardial infarction in mice [19]. Our previous studies also showed that genetic deficiency of DPP4 in rats improved cardiac function during endotoxemia and ischemia/reperfusion, which were partly associated with GLP-1 signaling [11],[20]. Furthermore, inhibition of DPP4 enzyme activity modulates the activity of several peptides such as chemokines, neuropeptide Y, and stromal cell derived factor-1 (SDF-1) via non–GLP-1 mechanisms of action [21], [22]. Evidences also proved that DPP4-deficient-based cytoprotection is more complex than GLP-1 signaling. G-CSF administration in combination with DPP4 inhibitor leads to the stabilization of active SDF-1, which attracted stem cells to the heart and improved outcome after myocardial infarction [23].

It remains unclear how deficiency of DPP4 leads to the protection of myocardium in animal experiments. In vitro cellular experiments should be performed to determine whether the decrease in DPP4 activity simply augmented the GLP-1 protective signaling pathway or that such decrease resulted in a change of cellular function. Several studies used neonatal cardiomyocytes or cardiac cell lines for in vitro cellular studies, but no study has examined the effects of GLP-1 or DPP4 inhibitor on adult cardiomyocytes. Accordingly, we isolated cardiomyocytes from two kinds of adult rats, examined, and compared their response to the reactive oxygen species (ROS) stress. ROS are thought to serve as a mediator in several cardiovascular disease [24]. Elevation of ROS results in potentially cytotoxic oxidative stress, which leads to apoptosis [24]. H_2_O_2_ is commonly used as an exogenous source of ROS, which increases lipid peroxidation, decreases antioxidant enzyme activity and induces cell injury in cardiomyocyte [25]. For these reasons, we investigated the responses of H_2_O_2_-induced oxidative stress in adult wild-type and DPP4-deficient rat isolated cardiomyocytes. The coadministration of GLP-1 or DPP4 inhibitor was also performed to define the mechanism.

## Materials and Methods

### 1. Experimental animals

The investigation was performed in accordance with the Guide for the Care and Use of Laboratory Animals published by the US National Institutes of Health (NIH publication no. 85–23, revised 1996), and was approved by the Institutional Animal Care and Use Committee of the National Taiwan University. Adult Fischer 344 and DPP4-deficient mutant (DchcHsd-DPP IV) rats were obtained from National Laboratory Animal Center of Taiwan and Harlan laboratories, Inc, respectively. Fischer 344 is the wild-type strain for the DPP4-deficient rats. Animals were maintained under a 12-h light/dark cycle at a controlled temperature (21±2°C) with free access to food and tap water.

### 2. Determination of DPP4 activity

Gly-Pro-AMC (St. Louis, MO, USA), a synthesis substrate of DPP4, was used to evaluate DPP4 activity in protein as described previously [11]. The amount of AMC liberation was determined for DPP4 activity. Cardiac protein in 50 mM HEPES solution containing 100 uM Gly-Pro-AMC was incubated at 37°C for 60 min, and the liberation of free AMC was monitored at 370 nm excitation and 440 nm emission.

### 3. Adult rat cardiomyocyte isolation

Adult ventricular cardiomyocytes were isolated from both Fischer 344 and DPP4-deficient rats. Rats were anesthetized with pentobarbital (50 mg/kg, i.p.) for 15 minutes. After rats appeared calm, hearts were quickly excised, and directly perfused on a modified Langendorff perfusion apparatus with Krebs buffer (containing: 110 mM NaCl, 2.6 mM KCl, 1.2 mM KH_2_PO_4_, 1.2 mM MgSO_4_, 25 mM NaHCO_3_ and 11 mM glucose (pH 7.4)) for 5 minutes. Buffer solutions were replaced by enzyme mixtures containing 0.04% w/v collagenase (type II, Washington Biochemical Corp.) and 0.02% w/v hyaluronidase for 20 minutes. Then, hearts were dissected mechanically to small pieces and shaken in flasks with enzyme mixture for another 40 minutes. The collagenase digested ventricle suspensions were then filtered through a mesh to remove remnants of connective tissues. After rinsing with solutions, cell suspensions were placed on top of BSA solution where non-cardiomyocytes were removed by a density gradient. Cells were cultured in medium with Dulbecco’s Modified Eagle’s Medium (DMEM) supplemented with antibiotics (100 µg/ml penicillin and 100 µg/ml streptomycin) at 37°C under a 5% CO_2_−95% air atmosphere. One hour after plating, the culture medium was changed to remove unattached dead cells and the viable cardiomyocytes were incubated overnight. After the initial incubation period, quiescent cardiomyocytes were treated with H_2_O_2_ (300 µM) for one hour. In some study, GLP-1 (200 nM) was preincubated for 60 min before H_2_O_2_ administration. To explore the mechanisms activated by GLP-1, antagonists of the GLP-1 receptor (Ex(9–39); 150 nM), PI3K (LY294002; 15 µM), MEK (PD98059; 15 µM), and PKA (H89; 20 µM) were used for 30 min before the addition of GLP-1. To evaluate whether loss of DPP4 activity induce protective effect against H_2_O_2_–induced ROS stress, a DPP4 inhibitor, diprotin A (50 µM), was added 90 min prior to H_2_O_2_ treatment.

### 4. Detection of cell viability

Cell viability was determined by MTT assay. Cells were treated with MTT (3-(4,5-Dimethylthiazol-2-yl)-2,5-diphenyltetrazolium bromide) at 0.5 mg/ml. The purple formazan crystals were dissolved in DMSO. Solutions were then loaded in a 96 well plate, and determined on an automated microplate spectrophotometer at 570 nm. Each condition tested was performed in triplicate in each experiment.

### 5. Detection of endogenously produced reactive oxygen species (ROS) in cardiomyocytes

ROS generation in cardiomyocytes was detected by labeling with fluorescence dye CM-H2DCFDA (5-(6)-chloromethyl-2′7′-dihydrofluorescein diacetate). By using fluorescence microscopy, cardiomyocyte ROS level could be monitored at 488 nm excitation and 515 nm emission. Fluorescence intensity was calculated by averaging fluorescence intensity of numerous outlined cells using Imagequant (Molecular Dynamics, Inc., Sunnyvale, CA, USA).

### 6. Detection of catalase activity in cardiomyocyte

Catalase activity was measured by ELISA kit (Cayman Chemicals, Ann Arbor, MI). Cardiomyocyte protein was prepared to measure catalase activity. After the addition of reaction mixture, the absorbance of each sample was read at 540 nm by using a 96-well microplate spectrophotometer. Each condition tested was performed in duplicate in each experiment.

### 7. Detection of glucose uptake in cardiomyocyte

Glucose uptake was measured by ELISA kit (Cayman Chemicals, Ann Arbor, MI). Fluorescently-tagged glucose derivative (2-NBDG) uptake was monitored at 485 nm excitation and 535 nm emission. Each condition tested was performed in triplicate in each experiment.

### 8. Detection of cell shortening in cardiomyocyte

Contractile properties of cardiomyocyts were measured by using an IonOptix myocyte contractility system (Ionoptix, Milton, MA USA). Cells were paced at 1 Hz via a pair of electrode to provide field stimulation. Only clear edges with rod shapped cardiomyocytes without spontaneous contraction were selected. Several parameters were chosen for analysis: maximal velocities of shortening/relengthening (±dL/dt), cell length of peak shortening, and time to peak shortening.

### 9. Protein extraction of cardiomyocyte

RIPA buffer (Tris–HCl 50 mM, NaCl 150 mM, EGTA 1 mM, EDTA 1 mM, NP-40 1%, sodium deoxycholate 1%) containing cocktail protease and phosphatase inhibitor (Sigma, St. Louis, MO, USA) was added to extract the protein of left ventricular or cardiomyocyte. Protein concentrations were determined by BCA protein assay kit (Thermo Fisher Scientific Inc., Rockford, IL, USA).

### 10. Western blot

Protein samples were denatured for 10 min in boiling sample buffer (31.3 mM Tris-HCl pH 6.8, 25% glycerol, 10% SDS, 10% 2-Mercaptoethanol, 0.00125% bromophenol blue). Proteins were separated by SDS-PAGE and transferred to polyvinylidene difluoride membranes (Perkin-Elmer Life Sciences, Boston, MA, USA). The membranes were blocked in 5% fat-free milk dissolved in TBST (Tris/phosphate/saline/Tween) and incubated with primary antibody to actin (purchased from Santa Cruz Biotechnology, Inc.), p-AKT (Ser-473), AKT, p-ERK (Thr-202/Tyr-204), ERK, Bax, Bcl-2 (purchased from Cell Signaling Technology, Inc) overnight at 4°C. The membranes were then washed several times with TBST and followed by further incubation with horseradish peroxidase-conjugated secondary antibody (Santa Cruz Biotechnology, Inc.) for an hour. After being washed several times, the signals were detected with the ECL system (Millipore, Bedford, MA, USA). The blots were scanned and quantified by Imagequant (Molecular Dynamics, Inc., Sunnyvale, CA, USA).

### 10. Statistical analysis

All values were presented as means ± SE. The results were analyzed using ANOVA followed by Bonferroni's post hoc tests. P<0.05 was considered as significant difference.

## Results

### 1. DPP4 activity in wild-type and DPP4-deficient heart

**Figure 1:**
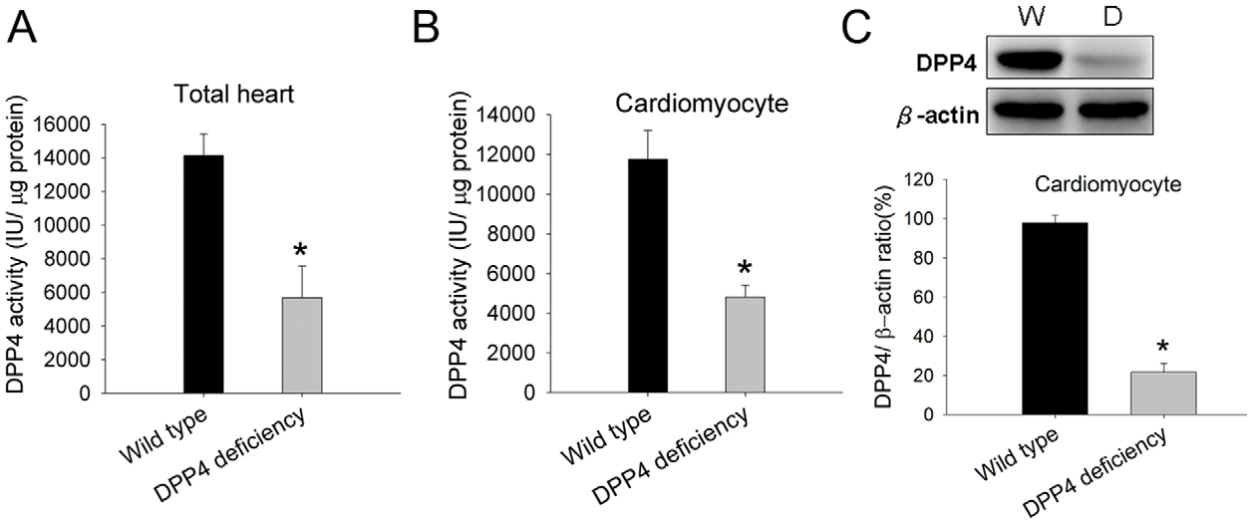
Measurement of DPP4 activity and protein expression. DPP4 activity of (A) total heart and (B) cardiomyocyte protein were measured in wild-type and DPP4-deficient rats. (C)DPP4 expression was measured in two kinds of cardiomoyocytes by using western blot. W indicates wild-type, and DPP4 indicates DPP4 deficiency. (n = 5) *p<0.05 vs. wild-type.

To confirm the deficiency of DPP4 activity in mutant (DchcHsd-DPPIV-) heart, DPP4 activity in both total heart and cardiomyocyte protein were measured (Fig. 1 A and B). The expression of DPP4 was determined by western blot (Fig 1C). DPP4 mutant is regarded as DPP4 deficiency, since both DPP4 expression and activity of isolated cardiomyocytes from mutant rats was much lower than that in wild-type rats.

### 2. Cell viability in DPP4-deficient and wild-type cardiomyocytes after H_2_O_2_ exposure

**Figure 2:**
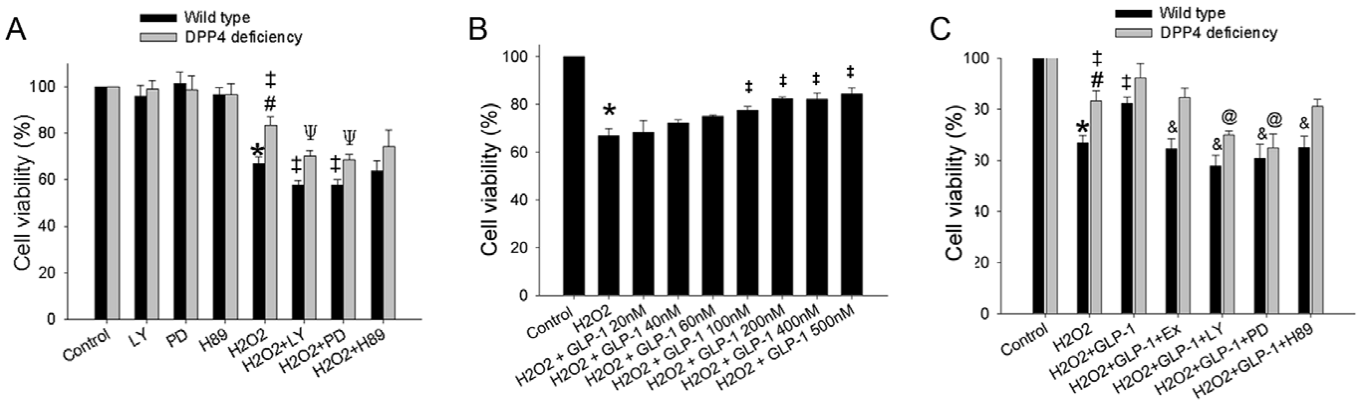
Effects of H_2_O_2_ treatment on cell viability in wild-type and DPP4-deficient cardiomyocytes. (A) Cardiomyocytes isolated from two kinds of rats underwent H_2_O_2_ (300 µM) treatment for an hour. (B) GLP-1 concentration ranging from 20 nM to 500 nM was preincubated for an hour before H_2_O_2_ administration in wild-type cardiomyocyte. (C) GLP-1 (200 nM) was added for an hour before H_2_O_2_ exposured in both wild-type and DPP4-deficient cardiomyocytes. Antagonists of the GLP-1 receptor (Ex(9–39); 150 nM), PI3K (LY294002; 15 µM), MEK (PD98059; 15 µM), and PKA (H89; 20 µM) were used for 30 min before the addition of GLP-1. Cell viability was tested via MTT assay. (n = 5). *p<0.05 vs. wild-type control, #p<0.05 vs. DPP4-deficient control, ‡p<0.05 vs. wild-type H_2_O_2_, Ψp<0.05 vs. DPP4-deficient H_2_O_2_, &p<0.05 vs. wild-type H_2_O_2_ + GLP-1, @p<0.05 vs. DPP4-deficient H_2_O_2_ + GLP-1.

To determine whether DPP4 deficiency exerted protective effect is directly or indirectly related to GLP-1, cardiomyocytes isolated from two kinds of rats underwent H_2_O_2_ treatment in the presence or absence of GLP-1 were examined (Fig. 2). Cell viability was then tested via MTT assay. Viability of DPP4-deficient cardiomyocytes was higher than that of wild-type cardiomyocytes after H_2_O_2_ exposure (Fig. 2A). We investigated the role of protein kinases PI3K, ERK, and PKA in the regulation of cell survival by using LY294002, PD98059, and H89, which are capable of selectively blocking PI3K, ERK, and PKA, respectively. In normal condition, the three inhibitors did not affect cell viability. On exposure to H_2_O_2_, PI3K and ERK inhibitors caused further reduction of cell viability in wild-type and DPP4-deficient cardiomyocytes. However, PKA inhibitor did not cause further reduction of cell viability in cardiomyocytes exposed to H_2_O_2_.

Since GLP-1 signaling was found to have a crucial role in protecting heart against I/R injury in DPP4-deficient rats [11], we investigated the role of GLP-1 in isolated cardiomyocytes. Different concentrations of GLP-1 were added one hour before wild-type cardiomyocyte exposed to H_2_O_2_ (Fig 2B). Treatment with GLP-1 was capable of improving cell viability. 40 nM of GLP-1 was shown to slightly exert protect effect, while 200 nM of GLP-1 reached the maximal response. We selected 200 nM of GLP-1 for the following study, since the ceiling response concentration may omit the augmentation of GLP-1 signaling in DPP4-deficient cardiomyocytes. Co-treatment with inhibitors of PI3K, ERK, PKA, and GLP-1 receptor antagonist reduced GLP-1 mediated protection of cell viability in wild-type cardiomyocyte (Fig. 2C). GLP-1 slightly but insignificantly improved cell viability in DPP4-deficient cardiomyocytes after H_2_O_2_ exposure.

### 3. H_2_O_2_-induced high ROS concentration was ameliorated in DPP4-deficient cardiomyocytes

**Figure 3:**
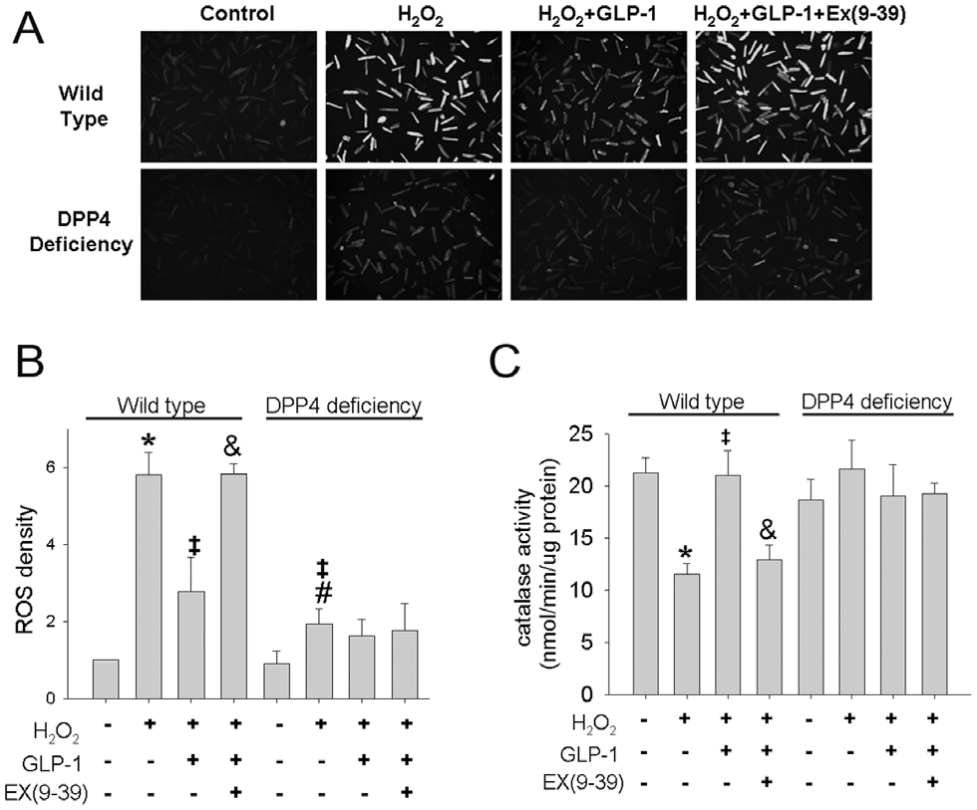
Effects of H_2_O_2_ treatment on ROS concentration and catalase activity in wild-type and DPP4-deficient cardiomyocytes. Cardiomyocytes isolated from two kinds of rats underwent H_2_O_2_ treatment in the presence or absence of GLP-1 or GLP-1 receptor antagonist, ex(9–39). (A) Original microscopy photos were reported for ROS density, and (B) results of densitometry. (n = 3) (C) Catalase activity was reported. (n = 5) *p<0.05 vs. wild-type control, #p<0.05 vs. DPP4-deficient control, ‡p<0.05 vs. wild-type H_2_O_2_, &p<0.05 vs. wild-type H_2_O_2_ + GLP-1.

The intracellular level of ROS was measured by loading isolated cardiomyocytes with CM-H2DCFDA. The original microcopy photo was shown (Fig. 3A), and the results were all quantified (Fig. 3B). Small amount of ROS was found in the control cardiomyocytes. After H_2_O_2_ treatment, the ROS level was significantly higher in the wild-type than that in the DPP4-deficient cardiomyocytes. GLP-1 pretreatment before H_2_O_2_ administration resulted in the alleviation of ROS concentraion in wild-type cardiomyocytes, and the protective effect of GLP-1 was shown to be diminished by ex(9–39), a GLP-1 receptor antagonist. However, GLP-1 failed to further decrease ROS level in DPP4-deficient cardiomyocytes. Catalase activity of wild-type cardiomyocyte was significantly decreased after H_2_O_2_ exposure (Fig. 3C). Pretreatment with GLP-1 significantly prevented the reduction of catalase activity after H_2_O_2_ exposure in wild-type cardiomyocytes, and the protective effect of GLP-1 was abolished by ex(9–39). Catalase activity in DPP4-deficient cardiomyocyte was not altered by H_2_O_2_. GLP-1 failed to change catalase activity of H_2_O_2_ exposed DPP4-deficient cardiomyocytes.

### 4. Bax/Bcl2 ratio and caspase-3 activity was lowered in DPP4-deficient cardiomyocytes after exposure to H_2_O_2_

**Figure 4:**
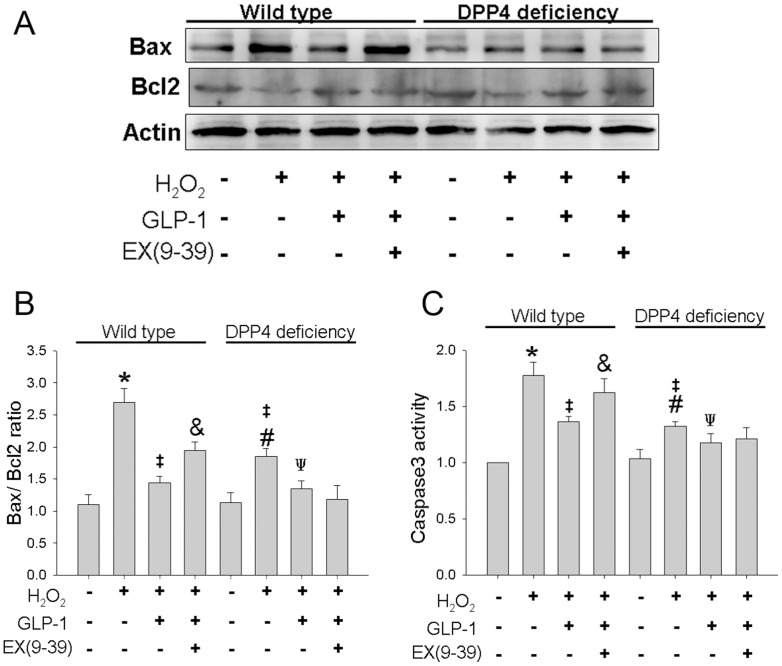
Effects of H_2_O_2_ treatment on Bax, Bcl-2 level, and caspase-3 activity in wild-type and DPP4-deficient cardiomyocytes, with or without GLP-1 or GLP-1 receptor antagonist cotreatment. (A) Original western blots of Bax and Bcl-2 were reported. (B) Ratios of Bax to Bcl-2, and (C) Caspase-3 activity were reported. (n = 4) *p<0.05 vs. wild-type control, #p<0.05 vs. DPP4-deficient control, ‡p<0.05 vs. wild-type H_2_O_2_, Ψp<0.05 vs. DPP4-deficient H_2_O_2_, &p<0.05 vs. wild-type H_2_O_2_ + GLP-1.

Bax/Bcl2 ratio serves as a marker to determine cell susceptibility to apoptosis. In the control condition, similar Bax/BcL2 ratios were found in both cardiomyocytes (Fig. 4A amd B). In response to H_2_O_2_, Bax/BcL2 ratio increased in wild-type cardiomyocytes. The elevation was less in DPP4-deficient cardiomyocytes. GLP-1 inhibited H_2_O_2_-induced Bax/Bcl2 ratio elevation in both cardiomyocytes, However, only GLP-1 induced reduction of Bax/Bcl2 ratio in wild-type cardiomyocyote was antagonized by ex(9–39).

Caspase-3 is a member of the cysteine-aspartic acid protease family. Sequential activation of caspase proteins plays a central role in cell apoptosis. Capase-3 activity was shown and found to be lower in DPP4-deficient cardiomyocytes than in wild-type cardiomyocytes after exposure to H_2_O_2_ (Fig. 4C). GLP-1 pretreatment before H_2_O_2_ administration resulted in the alleviation of capase-3 activity in both cardiomyocytes. Only GLP-1 induced alleviation of caspase-3 activity in wild-type cardiomyocytes was antagonized by ex(9–39).

### 5. p-ERK activation was upregulated in H_2_O_2_ treated cardiomcyoytes

**Figure 5:**
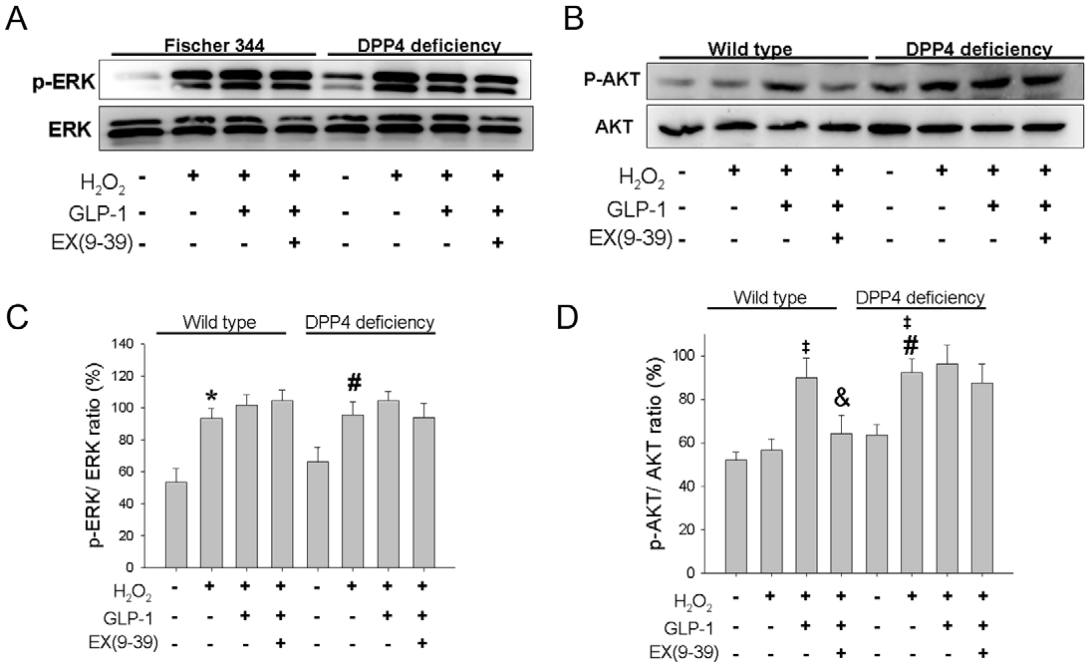
Effects of H_2_O_2_ treatment on ERK and AKT phosphorylation in wild-type and DPP4-deficient cardiomyocytes, with or without GLP-1 or GLP-1 receptor antagonist cotreatment. (A) Original western blots of ERK and p-ERK were reported. (C) Ratios of p-ERK/ERK were shown. (B) Original western blots of AKT and p-AKT were reported. (D) Ratios of p-AKT/AKT were shown. (n = 3–4) *p<0.05 vs. wild-type control, #p<0.05 vs. DPP4-deficient control, ‡p<0.05 vs. wild-type H_2_O_2_, &p<0.05 vs. wild-type H_2_O_2_ + GLP-1.

ERK protein phosphorylation appeared to be expressed similarly in control condition in both cardiomyocytes (Fig. 5A and C). Phosphorylation of ERK after H_2_O_2_ exposure was significantly increased in both cardiomyocytes, while cotreatment with GLP-1 or exendin(9–39) did not alter the expression.

### 6. p-AKT was activated in DPP4-deficient cardiomyocytes after exposure to H_2_O_2_

AKT protein phosphorylation appeared to be expressed similarly in control condition in both cardiomyocytes (Fig. 5B and D). Phosphorylation of AKT after H_2_O_2_ exposure was significantly increased in DPP4-deficient but not in wild-type cardiomyocytes. GLP-1 upregulated p-AKT after H_2_O_2_ treatment in wild-type cardiomyocytes, which was reversed by cotreatment with ex(9–39). GLP-1 did not further increase p-AKT level in DPP4-deficient cardiomyocytes.

### 7. DPP4 inhibitor did not improve cell viability in wild-type cardiomyocytes after H_2_O_2_ exposure

**Figure 6:**
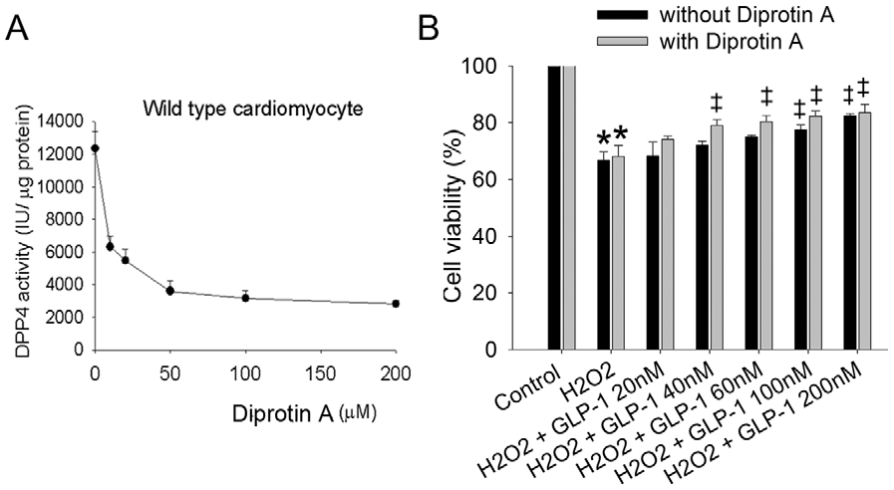
Effects of Diprotin A on cell viability after H_2_O_2_ treatment in wild-type cardiomyocyte. (A) DPP4 activity was measured in Diprotin A-treated wild-type cardiomyocyte. (B) Cardiomyocytes isolated from wild-type rats underwent H_2_O_2_ (300 µM) treatment for an hour in the absence or presence of Diprotin A (50 µM, 90 min prior to H_2_O_2_ treatment). GLP-1 was added for 60 min before the addition of H_2_O_2_. (n = 4) *p<0.05 vs. wild-type control, ‡p<0.05 vs. wild-type H_2_O_2_.

To find the appropriate concentration for diprotin A to inhibit DPP4 activity, wild-type cardiomyocytes were exposed to different concentrations of diprotin A (Fig. 6A). We selected 50 µM of diprotin A to inhibit DPP4 activity for the following study, and evaluate whether loss of DPP4 activity induces protective effect against ROS stress in wild-type cardiomyocytes. Diprotin A alone did not alter cell viability in wild-type cardiomyocytes exposed to H_2_O_2_ (Fig. 6B), but amplified the protective effect of low dose GLP-1 (40–100 nM). However, Diprotin A did not augment the protective effect of high dose GLP-1 (200 nM).

### 8. DPP4 deficiency and GLP-1 improved glucose uptake in cardiomyocytes

**Figure 7:**
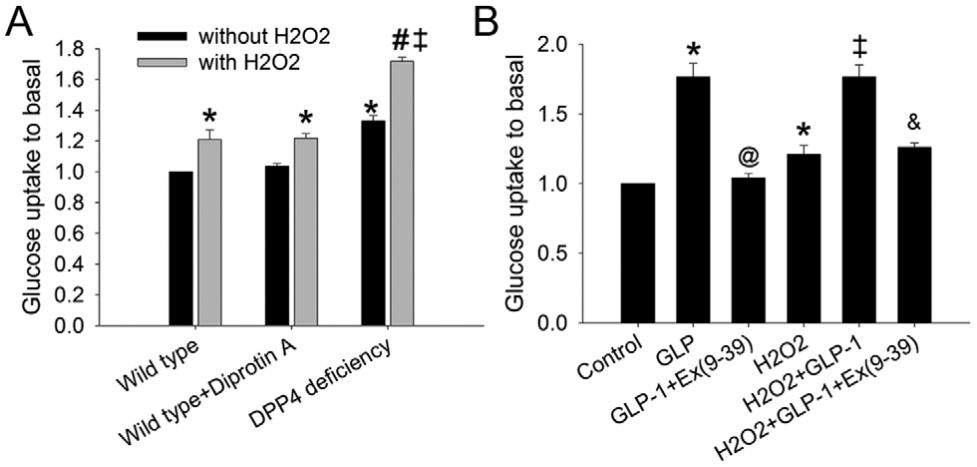
Effects of H_2_O_2_ treatment on glucose uptake in wild-type and DPP4-deficient cardiomyocytes. (A) Glucose uptake was measured in wild-type, wild-type + Diprotin A, and DPP4-deficient cardiomyocytes with or without H_2_O_2_ cotreatment. (B) Wild-type cardiomyocytes were treated with GLP-1 or GLP-1 + ex(9–39) in the absence or presence of H_2_O_2_, and along with glucose uptake measurement (n = 4). *p<0.05 vs. wild-type control, #p<0.05 vs. DPP4-deficient control, ‡p<0.05 vs. wild-type H_2_O_2_, &p<0.05 vs. wild-type H_2_O_2_ + GLP-1, @p<0.05 vs. wild-type + GLP-1.

To determine whether DPP4 deficiency affects energy production and fuel utilization, glucose uptake in cardiomyocytes were measured. Short term treatment of Diprotin A in wild-type cardiomyocytes did not alter glucose uptake in the control condition (Fig. 7A), but chronic DPP4 deficiency was shown to increase glucose uptake. Cardiomyocytes exposed to H_2_O_2_ resulted in increasing glucose uptake in each group, and a more prominent increase of glucose uptake was found in DPP4-deficient cardiomyocytes.

GLP-1 was shown to increase glucose transporter expression in myocardium [12], the effect of GLP-1 on glucose uptake was determined. GLP-1 increased glucose uptake in wild-type cardiomcyoytes in both control and H_2_O_2_–treated condition (Fig. 7B), and the effects were both abolished by ex(9–39), a GLP-1 receptor antagonist.

### 9. DPP4 deficiency and GLP-1 ameliorated contractile function after cardiomyocytes exposed to H_2_O_2_

**Figure 8:**
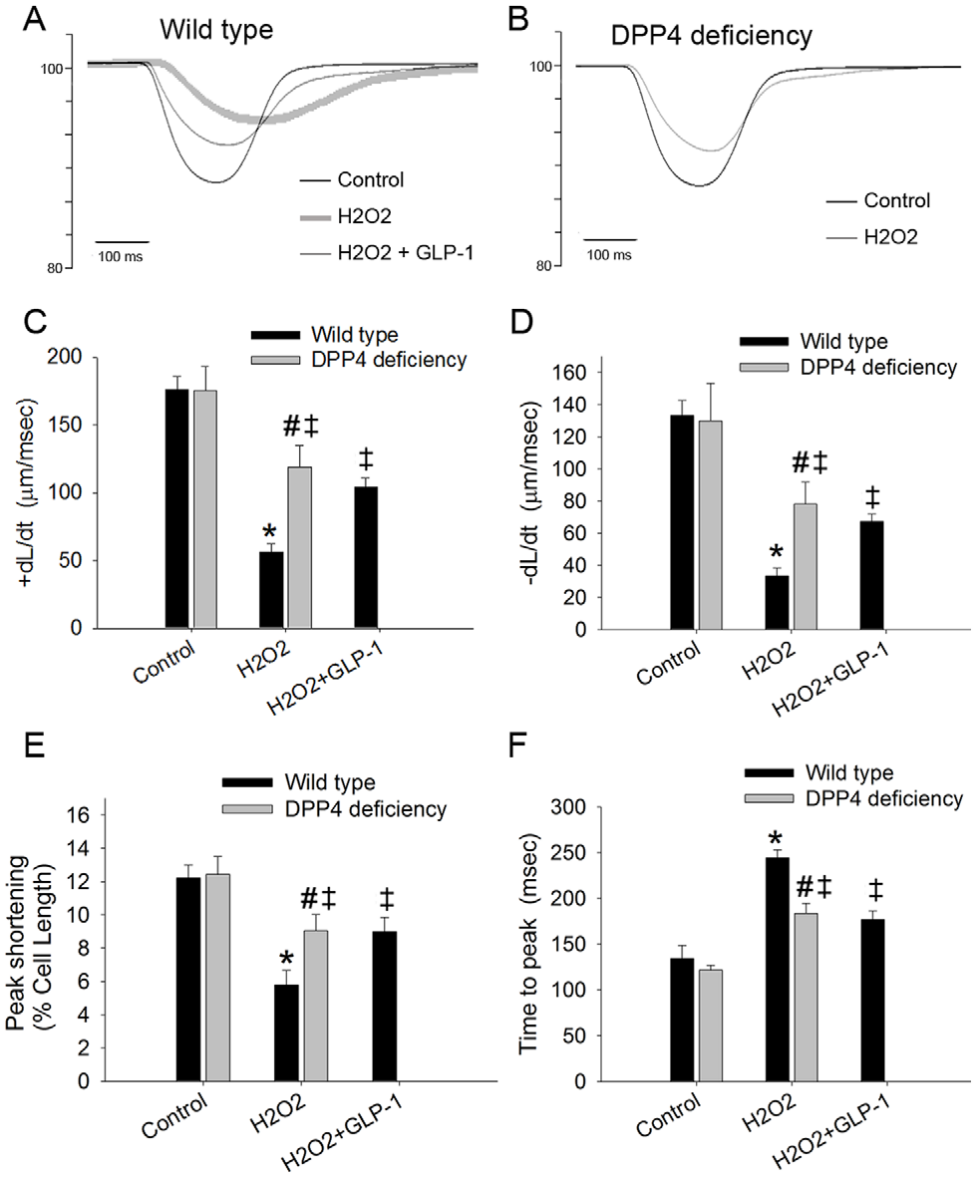
Effects of H_2_O_2_ treatment on contractile function in wild-type and DPP4-deficient cardiomyocytes. Cardiomcyoytes contractile function were measured after H_2_O_2_ (300 µM) exposure for an hour. GLP-1 (200 nM) was preincubated for an hour before H_2_O_2_ administration in wild-type cardiomyocytes. (A and B) Representative recordings of cell shortening were reported in wild-type and DPP4-deficient cardiomyocytes. (C) Maximal velocities of shortening (+dL/dt), (D) maximal velocities of relengthening (−dL/dt), (E) cell length of peak shortening, and (F) time to peak shortening were calculated. n = 22–30 cells from three rats per group. *p<0.05 vs. wild-type control, #p<0.05 vs. DPP4-deficient control, ‡p<0.05 vs. wild-type H_2_O_2_.

In order to understand whether the improvement of cell viability after H_2_O_2_ exposure affect contractile function, the cell shortening of cardiomyoyctes were performed. In the control condition, similar contractile function was found in both cardiomyoyctes (Fig. 8). Contractile function was suppressed after H_2_O_2_ exposure, with the reduction of peak shortening, ±dL/dt, and the prolongation of time to peak shortening. The attenuation of contractile function was more deteriorated in wild-type than that in DPP4-deficient cardiomyocytes. These H_2_O_2_-induced changes in wild-type cardiomcyoytes were partly prevented by GLP-1 treatment.

## Discussion

The present study demonstrated that H_2_O_2_ diminished catalase activity and increased intracellular ROS concentration, which led to increased Bax/Bcl-2 ratio, caspase-3 activation, and cell death. GLP-1 was shown to ameliorate H_2_O_2_-induced cell death in wild-type cardiomyocyte via preserving the activity of catalase and increasing the phosphorylation of AKT and glucose uptake, which were abolished by ex(9–39), suggesting the importance of GLP-1 receptor dependent pathway. The present study proved that DPP4-deficient cardiomyocytes were more resistant to H_2_O_2_ than wild-type cardiomyocytes in the absence of GLP-1 cotreatment. Furthermore, DPP4-deficient cardiomyocytes showed enhanced AKT phosphorylation, glucose uptake, and preserved catalase activity after H_2_O_2_ exposure. The improvement of cell viability after H_2_O_2_ exposure was correlated with the alleviation of cellular contractile dysfunction in both DPP4-deficient and GLP-1 treated wild-type cardiomyocytes. The results were consistent with our previous animal model experiments, that the cardiac performance after I/R injury in DPP4-deficient rats was better than that in wild-type rats [11]. DPP4 deficiency increased the capability against stress in both in vivo and in vitro studies, which were mediated more than GLP-1 dependent pathway

Cardiac energy metabolism is altered during stress. Enhancing the use of glucose as a fuel is beneficial to allow the heart to produce energy in ischemic condition [26], while stimulation of cardiomyocytes with H_2_O_2_ resulted in a concentration-dependent increase of glucose transporter expression [27]. Acute treatment of DPP4 inhibitor only augmented the protective effect of low dose GLP-1, but failed to improve cell viability alone in wild-type cardiomyocyte after H_2_O_2_ exposure. In addition, only long term but not acute inhibition of DPP4 activity increased glucose utilization in cardiomyocytes, indicating that chronic loss of DPP4 activity may induce functional change and increase capability of cardiomyocytes against H_2_O_2_ stress. The finding coincides with the other study that direct infusion of DPP4 inhibitor before ischemia did not improve functional recovery after I/R injury in the isolated heart [19], suggesting that acute reduction of cardiac DPP4 activity is not sufficient to produce cardioprotection. Indeed, ten days of treatment with sitagliptin enhanced the circulating levels of the GLP-1, and upregulated GLUT4 translocation and expression in spontaneously hypertensive rats hearts [28]. GLP-1 increased glucose uptake in perfusion heart, and increased GLUT4 translocation in animal model of dilated cardiomyopathy [12], [29]. Long term loss of DPP4 activity leads to the accumulation of plasma GLP-1 level in DPP4-deficient rats [11], [28], which may be partly associated with the improvement of glucose uptake in cardiomyocytes. Chronic deficiency of DPP4 activity may improve glucose utilization, which may therefore result in a resistance of cardiomyoyctes to oxidative stress.

Activation of AKT may preserve cardiac function during oxidative stress, and reduce myocyte apoptosis [30], [31], [32]. Inhibition of AKT signaling by PI3K/AKT inhibitor or genetic deletion abrogated the protective effect (Mullonkal and Toledo-Pereyra 2007). Activation of PI3K by GLP-1 was required to prevent proapoptotic events exposed to H_2_O_2_ in several kinds of culture cell [3], [33]. GLP-1 receptor agonist was shown to activate PI3K/AKT signaling to improve cell viability after hypoxia/reoxygenation treatment in isolated neonatal cardiomyocyte [6]. In our study, activation of PI3K was required to prevent cell death in response to H_2_O_2_. Elevation of p-AKT was associated with the amelioration of cell injury, while PI3K inhibitor reduced p-AKT and caused reduction of cell viability in both DPP4-deficient and GLP-1 treated wild-type cardiomyocytes. Since AKT pathway is involved in H_2_O_2_-mediated GLUT4 translocation and glucose uptake stimulation [27], [34], AKT activation in GLP-1 pathway and DPP4 deficiency may contribute to enhance glucose uptake and increase the resistance to oxidative stress. GLP-1 activated AKT signaling through its receptor-dependent pathway, but failed to cause further activation of AKT in DPP4-deficient cadiomyocytes after H_2_O_2_ exposure. We can not detect any auto-release of GLP-1 in cultured medium of DPP4-deficient cardiomyocytes (data not shown), and the mechanisms involved in the activation of AKT in DPP4-deficient cardiomyocytes remains unclear. DPP4 is known to exert its effects via both enzymatic and non-enzymatic mechanisms [35]. The non-enzymatic function of DPP4 may also regulate inflammatory responses in immune system, and its inhibition may have a role in the treatment of inflammatory diseases such as atherosclerosis [35]. Long term deficiency of DPP4 may modulate inflammatory response in cardiomyocytes, and may contribute to the AKT activation in response to oxidative stress.

ROS trigger the activation of multiple signaling pathways, instead of AKT pathway, ERK pathway is also induced in response to oxidative stress and plays an important role in regulating cell survival [36]. Several studies have shown that ERK activation prevents cell injury induced by H_2_O_2_ [37], [38]. In our study, H_2_O_2_ treatment caused a marked activation of ERK, while cotreatment with PD98059, a MEK kinase inhibitor, resulted in losing of cell viability, implying that ERK is an important survival pathway in cardiomyocyte under oxidative stress. Although GLP-1 or DPP4 deficiency did not alter the expression of p-ERK, ERK signaling can not be excluded in the GLP-1 and DPP4 defiiciency signaling for counteracting oxidative stress in cardiomyocyte. In fact, GLP-1 receptor agonist was shown to exert protective actions against hypoxia/reoxygenation induced cell death in isolated neonatal cardiomyocyte via ERK-dependent pathway [6]. GLP-1 also mediates antiapoptotic effect by phosphorylating Bad through a β-Arrestin 1-mediated ERK1/2 activation in pancreaticβ-cells [39].

GLP-1 receptor can trigger the generation of the second messenger cAMP and followed by activation of PKA [40]. Our data supported the importance of GLP-1 receptor signaling pathway in the alleviation of cell apoptosis after H_2_O_2_ exposure, by preserving catalase activity, alleviating of ROS level, Bax/Bcl2 ratio, and caspase-3 activity. The protective effect of GLP-1 is PKA-dependent, since the improvement of cell viability of GLP-1 after H_2_O_2_ exposure in wild-type cardiomyocyte was blocked by H89, a PKA inhibitor. In correlated with other studies, GLP-1 was shown to have an anti-apoptotic action mediated by a cAMP/PKA -dependent signaling pathway in pancreatic βcell and endothelial cell [3], [5]. In addition, activation of PI3K/AKT can be achieved by activation of PKA [41], and a direct interaction of PKA and AKT was also found in GLP-1 action [3]. GLP-1 receptor antagonist was shown to abolish the elevation of AKT phosphorylation in response to H_2_O_2_ in wild-type cardiomyocyte. Although the activation of AKT by PKA was not completely understood in the present study, our data favor the idea that GLP-1 receptor-PKA-AKT activation pathway is required in the protective signaling of GLP-1 in wild-type cardiomyocytes. However, it has been shown that GLP-1 improved cardiac function after I/R in GLP-1 receptor knock-out Langendorff-perfused heart [29]. An inactive cleaved form of GLP-1, GLP-1 (9–36), was also shown to have an anti-apoptotic effects in cardiomyocytes and endothelial cells in a alternative receptor [6], [42], [43]. The role of GLP-1 (9–36) can not be excluded in this study.

Our limitation of the study is that DPP4-deficient cardiomyocytes retained one third level of DPP4 activity, which is similar to that reported in a recent paper [44]. The maximal inhibition concentration of ditropin A also retained one third level of DPP4 activity. There are several subtypes of DPP, which may also cleavage Gly-Pro-AMC, the synthetic substrate of DPP4 [45]. The non-specific cleavage of Gly-Pro-AMC can not be excluded in the DPP4 activity study.

In conclusion, GLP-1 exerted protective effect against ROS stress via receptor-dependent AKT pathway. Long term deficiency of DPP4 induces changes of cellular function, and increase the capability against oxidative stress via enhancing AKT signaling and glucose uptake in association with preserving catalase activity, probably via both GLP-1 receptor –dependent and -independent signaling.
